# T cell receptors employ diverse strategies to target a p53 cancer neoantigen

**DOI:** 10.1016/j.jbc.2022.101684

**Published:** 2022-02-03

**Authors:** Daichao Wu, Ragul Gowathaman, Brian G. Pierce, Roy A. Mariuzza

**Affiliations:** 1 W.M. Keck Laboratory for Structural Biology, University of Maryland Institute for Bioscience and Biotechnology Research, Rockville, Maryland, USA; 2 Department of Histology and Embryology, Hengyang Medical School, University of South China, Hengyang, Hunan, China; 3 Department of Cell Biology and Molecular Genetics, University of Maryland, College Park, Maryland, USA

**Keywords:** T cell receptor, cancer neoantigen, oncogene, MHC, X-ray crystallography, ACT, adoptive cell therapy, *K*_*D*_, dissociation constant, pMHC, peptide–MHC, REU, Rosetta energy units, SPR, surface plasmon resonance, TIL, tumor-infiltrating lymphocyte, TCR, T cell receptor, Vα, variable α, Vβ, variable β

## Abstract

Adoptive cell therapy with tumor-specific T cells can mediate durable cancer regression. The prime target of tumor-specific T cells are neoantigens arising from mutations in self-proteins during malignant transformation. To understand T cell recognition of cancer neoantigens at the atomic level, we studied oligoclonal T cell receptors (TCRs) that recognize a neoepitope arising from a driver mutation in the p53 oncogene (p53R175H) presented by the major histocompatibility complex class I molecule HLA-A2. We previously reported the structures of three p53R175H-specific TCRs (38-10, 12-6, and 1a2) bound to p53R175H and HLA-A2. The structures showed that these TCRs discriminate between WT and mutant p53 by forming extensive interactions with the R175H mutation. Here, we report the structure of a fourth p53R175H-specific TCR (6-11) in complex with p53R175H and HLA-A2. In contrast to 38-10, 12-6, and 1a2, TCR 6-11 makes no direct contacts with the R175H mutation, yet is still able to distinguish mutant from WT p53. Structure-based *in silico* mutagenesis revealed that the 60-fold loss in 6-11 binding affinity for WT p53 compared to p53R175H is mainly due to the higher energetic cost of desolvating R175 in the WT p53 peptide during complex formation than H175 in the mutant. This indirect strategy for preferential neoantigen recognition by 6-11 is fundamentally different from the direct strategies employed by other TCRs and highlights the multiplicity of solutions to recognizing p53R175H with sufficient selectivity to mediate T cell killing of tumor but not normal cells.

Adoptive cell therapy (ACT) with tumor-specific T cells can promote durable regression of diverse cancers, including metastatic melanoma, colon, bile duct, cervix, and breast cancers (1, 2, 3, 4, 5). The therapeutic effect of these tumor-infiltrating lymphocytes (TILs) is mediated primarily by cytotoxic CD8^+^ T cells (6). The main target of tumor-specific T cells are neoantigens that result from DNA alterations during malignant transformation (7). Of special interest are neoantigens derived from oncogenes bearing driver mutations because these mutations are tumor-specific, important for tumor progression, and generally expressed by all tumor cells (8). In a pioneering study of ACT, a patient with metastatic colorectal cancer was treated successfully with four *ex vivo*-expanded CD8^+^ T cell clones specific for a neoepitope arising from the G12D driver mutation in the KRAS oncogene (2, 9).

*TP53* (tumor protein p53) was the first tumor suppressor gene identified and is inactivated in the large majority of human cancers (10, 11). Mutations in *TP53* effect most of the hallmarks of cancer cells, including proliferation, genomic instability, and metastasis (12, 13). Hotspot positions include R175, G245, R248, R273, and R282, which cluster in the central DNA-binding domain of p53 and alter its DNA-binding properties (14). Mutations at these sites are attractive candidates for targeted immunotherapy because they confer a growth advantage to tumor cells and are associated with malignant progression.

The immunogenicity of p53 mutations in cancer patients has been demonstrated by the detection of T cell responses against several p53 neoantigens, most notably R175H in which arginine at position 175 is replaced by histidine (15, 16). This driver mutation is the most frequently observed mutation in *TP53* as well as the most common mutation in any tumor suppressor gene (17). A number of T cell receptors (TCRs) have been isolated from TILs of epithelial cancer patients that target a neoepitope corresponding to residues 168 to 176 of p53R175H (HMTEVVRHC; mutant amino acid in bold) (15, 16). The TCRs are restricted by HLA-A∗02:01, which is the most frequent major histocompatibility complex (MHC) class I allele in the U.S. population (18). These TCRs may prove effective in eliminating tumors expressing HLA-A2∗02:01 and the p53R175H mutation when transduced into a patient’s peripheral blood lymphocytes for ACT (15, 16).

With the aim of understanding TCR recognition of cancer neoantigens at the atomic level, we previously determined crystal structures of three p53R175H-specific TCRs (12-6, 38-10, and 1a2) in complex with HLA-A∗02:01 and the neoepitope p53R175H (19). The structures revealed that these TCRs discriminate between WT and mutated p53 by focusing on the R175H mutation, with which they make extensive interactions. Here, we report the structure of a fourth p53R175H-specific TCR (6-11) bound to the p53R175H peptide and HLA-A∗02:01. In sharp contrast to 12-6, 38-10, and 1a2, TCR 6-11 makes no contacts with the R175H mutation, yet is nevertheless able to distinguish mutant from WT p53. Collectively, these structures demonstrate that there are multiple distinct solutions to recognizing the p53R175H neoepitope with sufficient on-target affinity and specificity to mediate the killing of tumor cells expressing mutant p53 without affecting normal cells expressing WT p53, a critical consideration for avoiding adverse clinical events in ACT due to off-target TCR recognition (20).

## Results

### T cell receptor 6-11 discriminates between mutant and WT p53 peptides

**Figure 1:**
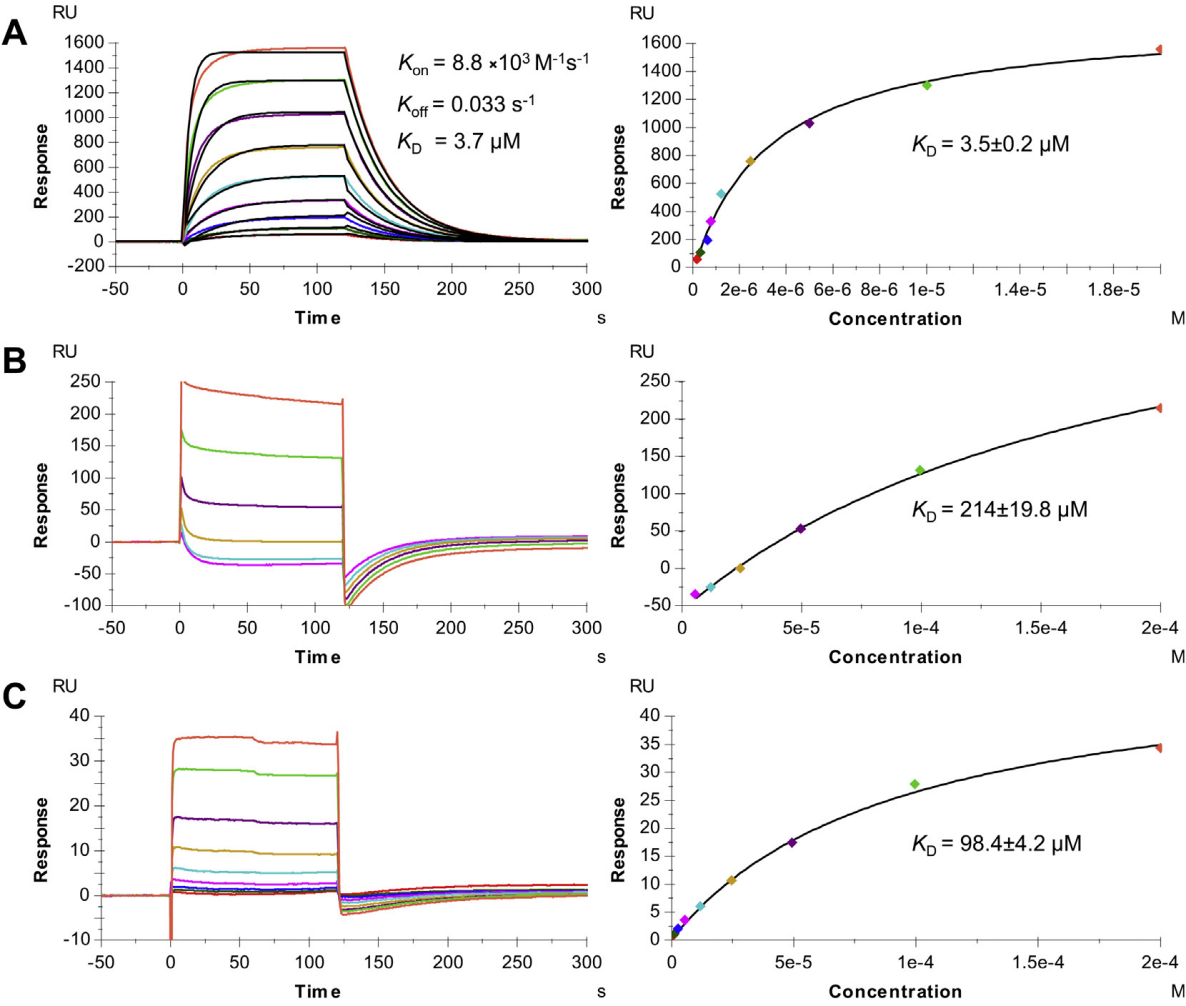
**Surface plasmon resonance analysis of TCR 6-11 binding to p53–HLA-A2 and p53R175H–HLA-A2.***A*, *left*, T cell receptor 6-11 at concentrations of 0.078, 0.156, 0.31, 0.625, 1.25, 2.5, 5, 10, and 20 μM was injected over immobilized p53R175H–HLA-A2 (3000 RU). The curves show kinetic fits. *Right*, fitting curve for equilibrium binding that resulted in a *K*_*D*_ of 3.5 ± 0.2 μM. *B*, *left*, T cell receptor 6-11 at concentrations of 6.25, 12.5, 25, 50, 100, and 200 μM was injected over immobilized p53–HLA-A2 (4000 RU). *Right*, fitting curve for equilibrium binding that resulted in a *K*_*D*_ of 214 ± 19.8 μM. *C*, *left*, T cell receptor 6-11 at concentrations of 0.78, 1.56, 3.12, 6.25, 12.5, 25, 50, 100, and 200 μM was injected over immobilized p53R175A–HLA-A2 (1000 RU). *Right*, fitting curve for equilibrium binding that resulted in a *K*_*D*_ of 98.4 ± 4.2 μM. All experiments were repeated three times. TCR, T cell receptor.

T cell receptor 6-11 was isolated by screening TILs from patients with metastatic colorectal cancer for reactivity toward the mutated p53R175H neoantigen (16). This HLA-A2∗0201-restricted TCR recognizes the p53R175H neoepitope using TRAV6 and TRAJ43 for the α chain and TRBV11-2 and TRBJ2-2 for the β chain. These gene segments are completely different from those utilized by TCRs 12-6, 38-10, and 1a2, which recognize the same p53R175H–HLA-A2 ligand as 6-11 (Table 1). We used surface plasmon resonance (SPR) to measure the affinity of TCR 6-11 for HLA-A2 loaded with mutant or WT p53 peptide (Fig. 1). Recombinant TCR 6-11 and peptide–MHC (pMHC) proteins were produced by *in vitro* folding from *Escherichia coli* inclusion bodies. Biotinylated p53R175H–HLA-A2 or p53–HLA-A2 was directionally coupled to a streptavidin-coated biosensor surface, and increasing concentrations of 6-11 were flowed sequentially over the immobilized pMHC ligand. T cell receptor 6-11 bound p53R175H–HLA-A2 with a dissociation constant (*K*_*D*_) of 3.5 ± 0.2 μM (Fig. 1*A*). This affinity is comparable to those of TCRs 12-6 (*K*_*D*_ = 1.1 μM), 38-10 (39.9 μM), and 1a2 (16.2 μM) (19). Kinetic parameters (on- and off-rates) for the binding of 6-11 to p53R175H–HLA-A2 were *k*_on_ = 8.8 × 10^3^ M^−1^s^−1^ and *k*_off_ = 0.033 s^−1^, corresponding to a *K*_*D*_ of 3.7 μM, in close agreement with the *K*_*D*_ from equilibrium analysis (3.5 μM). T cell receptor 6-11 bound WT p53–HLA-A2 with *K*_*D*_ = 214 ± 19.8 μM, which is ∼60-fold weaker affinity than for mutant p53R175H–HLA-A2 (Fig. 1*B*). By contrast, no apparent interaction could be detected between TCRs 12-6, 38-10, or 1a2 and WT p53–HLA-A2 (19). Thus, based on SPR, 6-11 is not as highly specific for mutant p53R175H–HLA-A2 as these other TCRs. In functional assays, T cells transduced with TCR 6-11 could be activated by antigen-presenting cells pulsed with subnanomolar concentrations of mutant p53R175H peptide, but they did not respond to WT p53 peptide, even at >1000-fold higher concentrations (16). Therefore, a *K*_*D*_ of 214 μM for 6-11 binding to p53–HLA-A2, although measurable by SPR, is below the affinity threshold required for the physiological activation of 6-11 T cells.

**Table 1:** Neoepitope p53R175H-reactive TCR germline genes and CDR3 sequences

Name	TRAV	TRAJ	CDR3α	TRBV	TRBJ	CDR3β	Reference
6-11	6	43	CALDIYPHDMRF	11-2	2-2	CASSLDPGDTGELFF	(15)
12-6	12-1	13	CVVQPGGYQKVTF	6-1	2-7	CASSEGLWQVGDEQYF	(15)
38-10	38-1	28	CAFMGYSGAGSYQLTF	10-3	1-6	CAISELVTGDSPLHF	(15)
1a2	12-3	12	CAMSGLKEDSSYKLIF	27	2-3	CASSIQQGADTQYF	(14)

### Overview of the 6-11–p53R175H–HLA-A2 complex

**Figure 2:**
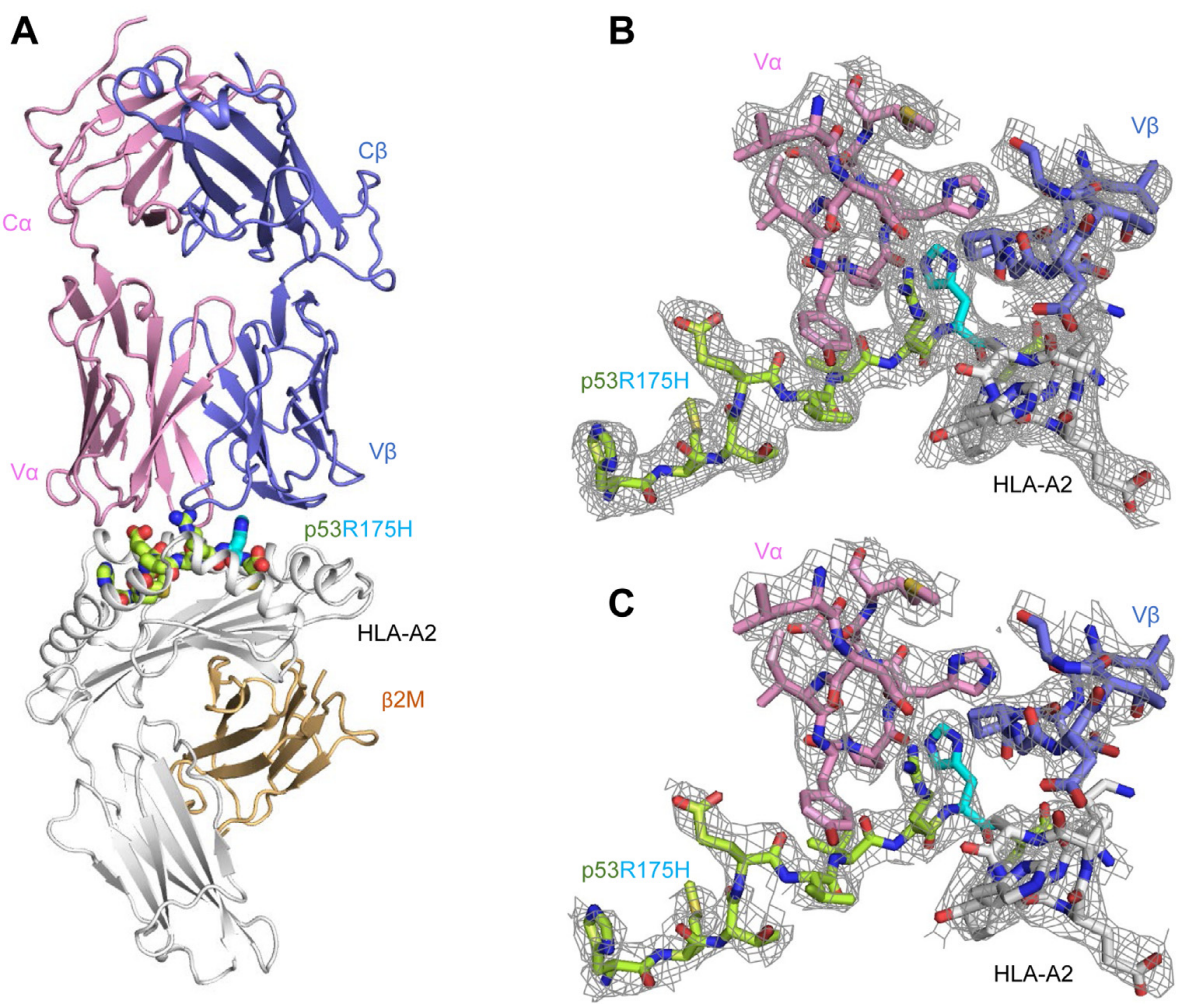
**Structure of the TCR 6-11–p53R175H–HLA-A2 complex.***A*, side view of the 6-11–p53R175H–HLA-A2 complex (*ribbon* diagram). TCR α chain, *pink*; TCR β chain, *blue*; HLA-A2 heavy chain, *gray*; and β_2_-microglobulin (β_2_m), *wheat*. The p53R175H peptide is *green* with the mutated P8 His residue highlighted in *cyan*. *B*, electron density in the interface of the 6-11–p53R175H–HLA-A2 complex. Density from the final 2*F*_o_ – *F*_c_ map at 3.33 Å resolution is contoured at 1σ. *C*, electron density in the complex interface. The *F*_o_ – *F*_c_ omit map at 3.33 Å resolution is contoured at 1σ. TCR, T cell receptor; Vα, variable α; Vβ, variable β.

To understand how TCR 6-11 discriminates between WT and mutant p53 epitopes, and to compare discrimination by 6-11 with that by 12-6, 38-10, and 1a2, we determined the structure of the 6-11–p53R175H–HLA-A2 complex to 3.33 Å resolution (Table S1) (Fig. 2*A*). The interface between TCR and pMHC was in unambiguous electron density for each of the four complex molecules in the asymmetric unit of the crystal (Fig. 2*B*). The rmsd in α-carbon positions for the TCR VαVβ and MHC α1α2 modules, including the p53R175H peptide, ranged from 0.18 Å to 0.35 Å for the four 6-11–p53R175H–HLA-A2 complexes, indicating close similarity. Therefore, the following description of TCR–pMHC interactions applies to all molecules in the asymmetric unit of the crystal.

**Figure 3:**
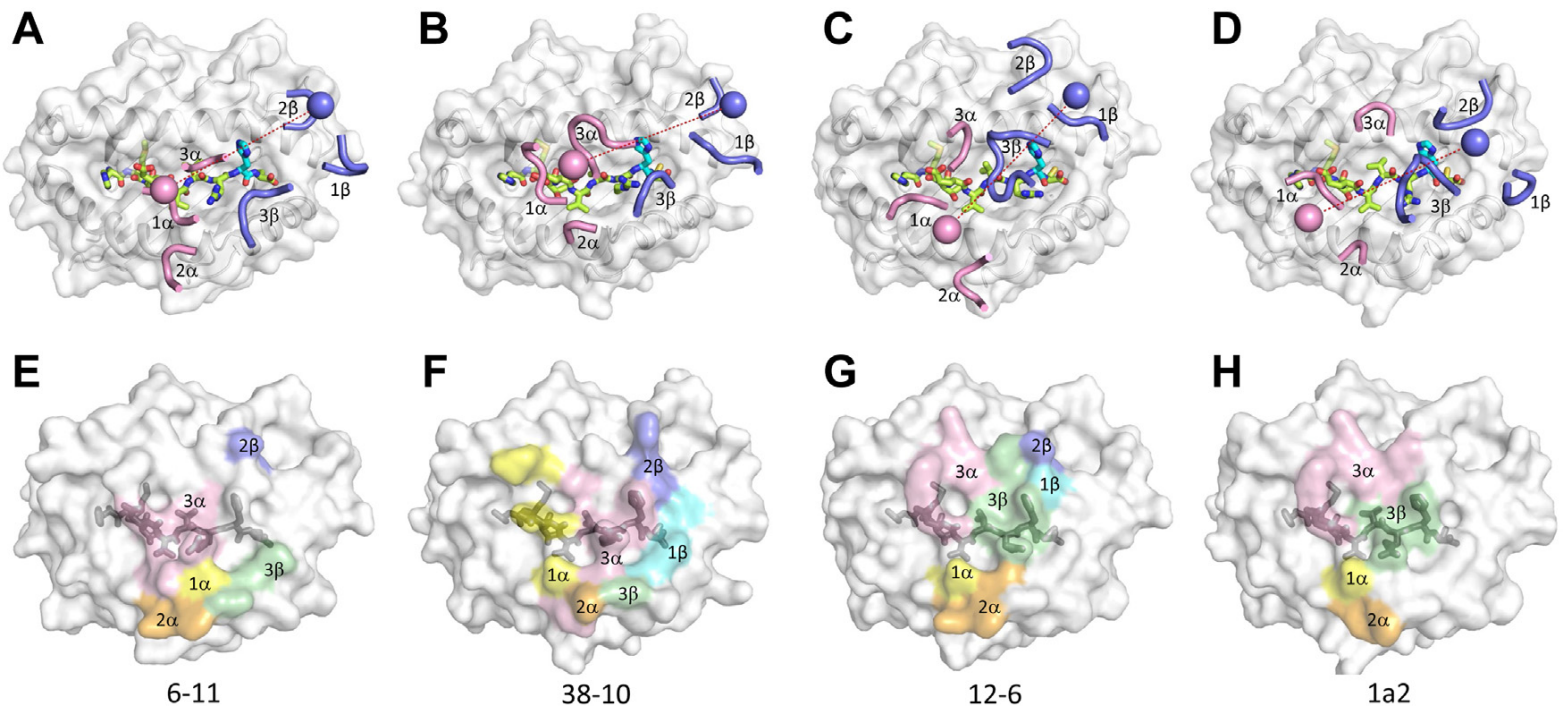
**Comparison of TCR footprints on p53R175H–HLA-A2.***A*, positions of CDR loops of TCR 6-11 on p53R175H–HLA-A2 (*top view*). CDRs of 6-11 are shown as numbered *pink* (CDR1α, CDR2α, and CDR3α) or *blue* (CDR1β, CDR2β, and CDR3β) loops. HLA-A2 is depicted as a *light gray surface*. The p53R175H peptide is drawn in *green stick* representation with the mutated P8 His residue in *cyan*. The *pink* and *light blue spheres* mark the positions of the conserved intrachain disulfide of the Vα and Vβ domains, respectively. The *red dashed line* indicates the crossing angle of TCR to pMHC. *B*, positions of CDR loops of TCR 38-10 on p53R175H–HLA-A2 (*top view*). *C*, positions of CDR loops of TCR 12-6 on p53R175H–HLA-A2 (*top view*). *D*, positions of CDR loops of TCR 1a2 on p53R175H–HLA-A2 (*top view*). *E*, footprint of TCR 6-11 on p53R175H–HLA-A2. The top of the MHC molecule is depicted as a *light gray surface*. The areas contacted by individual CDR loops are color-coded: CDR1α, *yellow*; CDR2α, *orange*; CDR3α, *pink*; CDR1β, *cyan*; CDR2β, *blue*; CDR3β, *green*. *F*, footprint of TCR 38-10 on p53R175H–HLA-A2. *G*, footprint of TCR 12-6 on p53R175H–HLA-A2. *H*, footprint of TCR 1a2 on p53R175H–HLA-A2. pMHC, peptide-MHC; TCR, T cell receptor; Vα, variable α; Vβ, variable β.

T cell receptor 6-11 docks over p53R175H–HLA-A2 in a canonical diagonal orientation, with variable α (Vα) over the α2 helix of HLA-A2 and variable β (Vβ) over the α1 helix. The crossing angle of TCR to pMHC (21) is 35°, which is similar to the crossing angles of 38-10 (34°) and 1a2 (30°) but more acute than that of 12-6 (51°) (Fig. 3, *A*–*D*). The incident angle (22), which corresponds to the degree of tilt of TCR over pMHC is 19° for 6-11, compared to 20° for 12-6, 27° for 38-10, and 1° for 1a2. Thus, the 6-11 complex is most like the 38-10 complex with respect to crossing angle and most like the 12-6 complex with respect to incident angle.

T cell receptor 6-11, like TCRs 12-6, 38-10, and 1a2 (19), is shifted toward the C-terminus of the p53R175H peptide, which is the site of the driver mutation at P8. To quantitate the shifts, we projected the positions of the TCR centers onto the pMHC plane, where the *x*-axis is aligned with the peptide and a more positive *x* value indicates a C-terminal shift (Table S2). Of note, 6-11 exhibits the seventh-highest C-terminal shift among 137 reported TCR–pMHC structures, which is nearly as much as 38-10 (third-highest) but more than 12-6 (23rd-highest) or 1a2 (27th-highest). The C-terminal shift of these TCRs is the key to their ability to discriminate between WT and mutant p53 peptides (see below).

As depicted by the footprint of TCR 6-11 on the pMHC surface (Fig. 3*E*), 6-11 establishes contacts with the p53R175H peptide mainly *via* the complementarity-determining region 3α (CDR3α) loop. Surprisingly, there are no contacts with P8 His, whose side chain represents the only structural difference between the mutant p53R175H–HLA-A2 and WT p53–HLA-A2 complexes (23). In sharp contrast to 6-11, TCRs 12-6, 38-10, and 1a2 all engage P8 His, either through CDR3α (38-10) or CDR3β (12-6 and 1a2) (Fig. 3, *F*–*H*). Overall, the footprint of 6-11 on pMHC mostly resembles that of 38-10 (Fig. 3, *E* and *F*), in agreement with the similar crossing angles and C-terminal shifts of these two TCRs, despite the usage of unrelated α/β chain pairs (Table 1).

### Interaction of TCR 6-11 with HLA-A2

**Figure 4:**
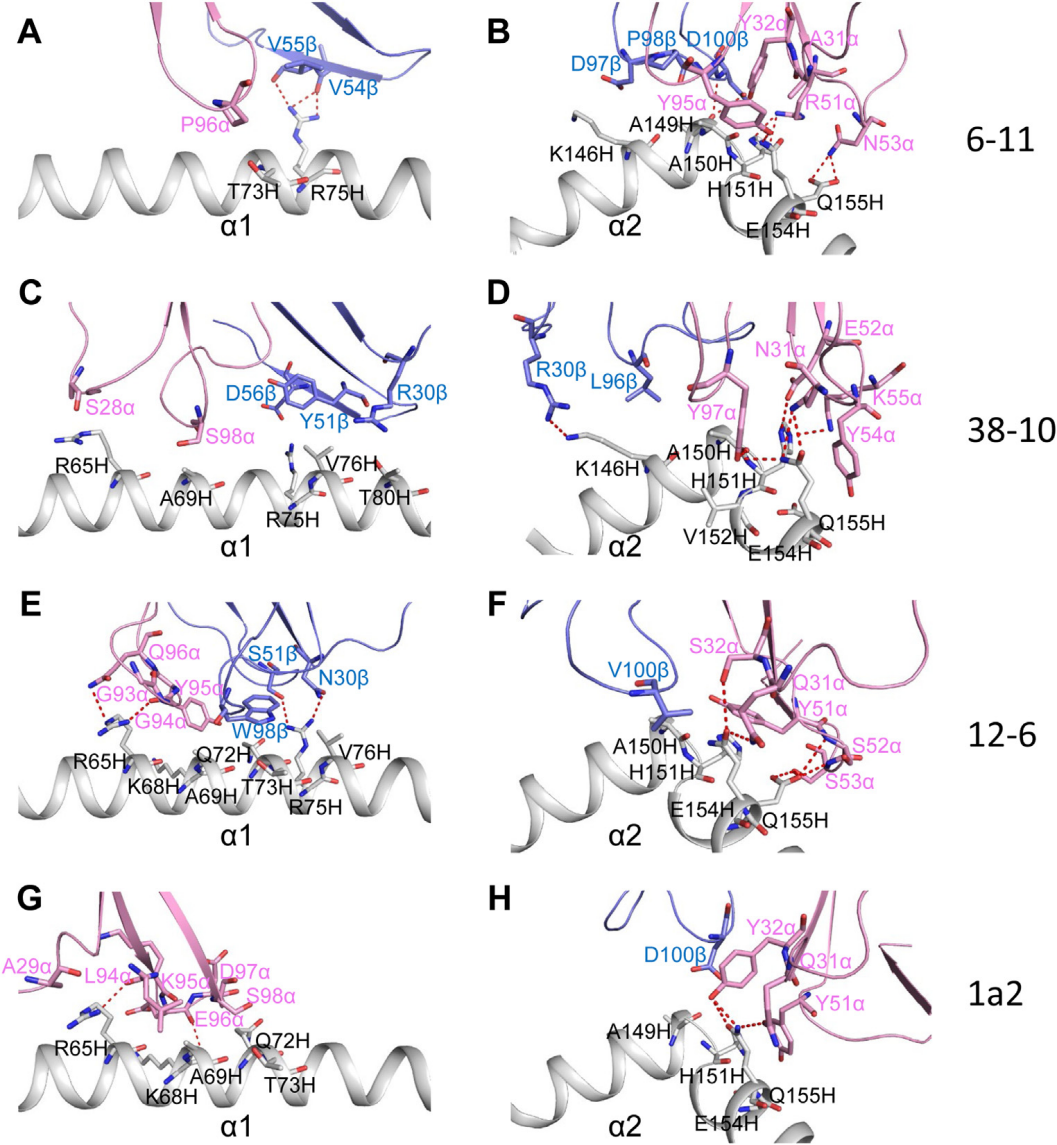
**Interactions of TCRs with HLA-A2.***A*, interactions between 6-11 and the HLA-A2 α1 helix. The side chains of contacting residues are drawn in *stick* representation with carbon atoms in *pink* (TCR α chain), *blue* (TCR β chain) or *light gray* (HLA-A2), nitrogen atoms in *dark blue*, and oxygen atoms in *red*. Hydrogen bonds are indicated by *red dashed lines*. *B*, interactions between 6-11 and the HLA-A2 α2 helix. *C*, interactions between 38-10 and the HLA-A2 α1 helix. *D*, interactions between 38-10 and the HLA-A2 α2 helix. *E*, interactions between 12-6 and the HLA-A2 α1 helix. *F*, interactions between 12-6 and the HLA-A2 α2 helix. *G*, interactions between 1a2 and the HLA-A2 α1 helix. *H*, interactions between 1a2 and the HLA-A2 α2 helix. TCR, T cell receptor.

T cell receptor 6-11 engages HLA-A2 through interactions distinct from those of 12-6, 38-10, or 1a2 (Fig. 4), but with some broad similarities. Of the total number of contacts (60) that 6-11 makes with HLA-A2, excluding p53R175H, CDR1α, CDR2α, and CDR3α contribute 13%, 33%, and 12%, respectively, compared with 0%, 15%, and 27% by CDR1β, CDR2β, and CDR3β, respectively (Table 2). Although Vα mediates more interactions with MHC than Vβ (35 of 60 contacts; 58%), it is not nearly as dominant in the 6-11–p53R175H–HLA-A2 complex as in the 38-10–p53R175H–HLA-A2, 12-6–p53R175H–HLA-A, and 1a2–p53R175H–HLA-A2 complexes, where Vα accounts for 74%, 77%, and 99%, respectively, of contacts with MHC (Table 2). The considerably fewer Vβ–MHC than Vα–MHC interactions in all four complexes is mainly due to the pronounced shift of the TCRs toward the C-terminus of the p53R175H peptide (Table S2), which partially disengages Vβ from the MHC α1 and α2 helices.

**Table 2:** TCR CDR atomic contacts with peptide and MHC

# of contacts		α chain				β chain				Total^a^
		CDR1	CDR2	HV4	CDR3	CDR1	CDR2	HV4	CDR3	
6-11	peptide	4	0	0	36	0	0	0	2	42
	MHC	8	20	0	7	0	9	0	16	60
38-10	peptide	12	0	0	54	11	1	0	4	82
	MHC	8	6	0	14	5	2	0	3	38
12-6	peptide	0	0	0	3	0	0	0	61	64
	MHC	5	39	0	28	5	3	0	12	92
1a2	peptide	11	0	0	13	0	1	0	36	61
	MHC	13	18	0	28	0	0	0	1	60

% of contacts		α chain				β chain			
		CDR1	CDR2	HV4	CDR3	CDR1	CDR2	HV4	CDR3
6-11	peptide	10	0	0	86	0	0	0	5
	MHC	13	33	0	12	0	15	0	27
38-10	peptide	15	0	0	66	13	1	0	5
	MHC	21	16	0	37	13	5	0	8
12-6	peptide	0	0	0	5	0	0	0	95
	MHC	5	42	0	30	5	3	0	13
1a2	peptide	18	0	0	21	0	2	0	59
	MHC	22	30	0	47	0	0	0	2

Contacts were calculated between nonhydrogen atoms with a 4.0 Å distance cutoff.

a Total contacts reflect the total number of TCR–MHC or TCR–peptide contacts.

T cell receptor 6-11 makes many more interactions with the HLA-A2 α2 helix than the α1 helix (Fig. 4, *A* and *B*), largely as a consequence of the moderately tilted binding mode of this TCR, which is characterized by an incident angle of 19°. In this respect, 6-11 resembles 38-10 (Fig. 4, *C* and *D*), which also makes only sparse contacts the HLA-A2 α1 helix, but differs from 12-6 (Fig. 4, *E* and *F*) and 1a2 (Fig. 4, *G* and *H*), which engage the α1 and α2 helices to similar extents (Table S3). 6-11 binds the HLA-A2 α2 helix using all three Vα CDR loops and Vβ CDR3. Thus, Arg51α, Asn53α, Tyr95α, and Asp100β form a cluster of seven hydrogen bonds with Ala149H, His151H, Glu154H, and Gln155H (Fig. 4*B*). Further anchoring 6-11 to HLA-A2 are three hydrogen bonds between Val54β and Val55β and Arg75H of helix α1. Each of these interactions, including those mediated by germline-encoded CDR2α and CDR2β residues, is unique to the 6-11–p53R175H–HLA-A2 complex (Fig. 4) (Table S3).

### Peptide recognition by TCR 6-11

**Figure 5:**
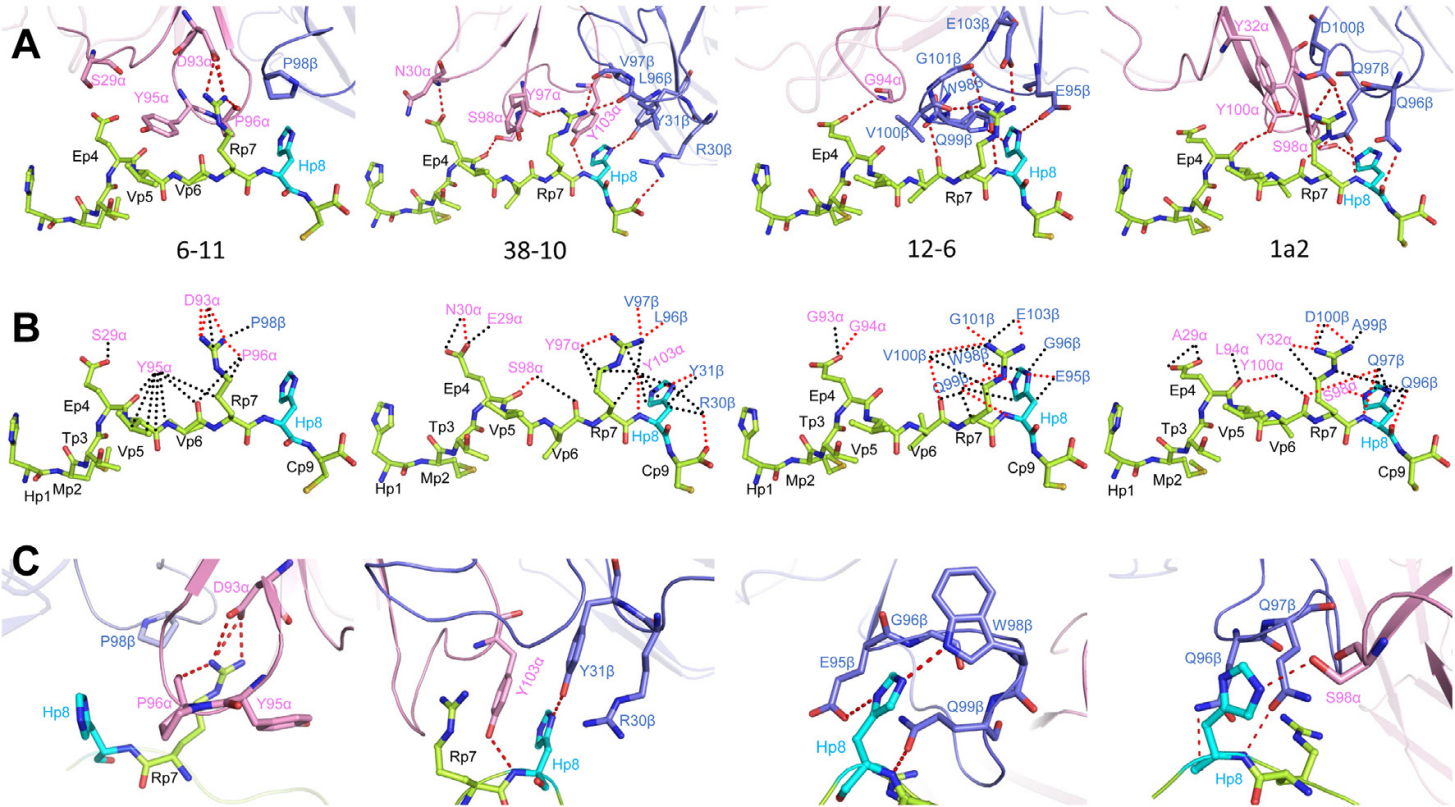
**Interactions of TCRs with the p53R175H peptide.***A*, interactions between 6-11, 38-10, 12-6, and 1a2 and the p53R175H peptide. The side chains of contacting residues are shown in *stick* representation with carbon atoms in *pink* (TCR α chain), *blue* (TCR β chain), *green* (p53R175H), or *cyan* (mutated P8 His), nitrogen atoms in *dark blue*, oxygen atoms in *red*, and sulfur atoms in *yellow*. Peptide residues are identified by one-letter amino acid designation followed by position (p) number. Hydrogen bonds are indicated by *red dashed lines*. *B*, comparison of interactions between 6-11, 38-10, 12-6, and 1a2 and the p53R175H peptide. Hydrogen bonds are *red dotted lines* and van der Waals contacts are *black dotted lines*. *C*, close-up of interactions between 6-11, 38-10, 12-6, and 1a2 and the p53R175H peptide. Contact residues were identified with CONTACT (31). Hydrogen bonds were calculated using a cut-off distance of 3.5 Å. The cut-off distance for van der Waals contacts was 4.0 Å. TCR, T cell receptor.

Upon binding p53R175H–HLA-A2, TCR 6-11 buries 67% (299 Å^2^) of the peptide solvent-accessible surface, compared to 71% (303 Å^2^) for 12-6, 76% (336 Å^2^) for 38-10, and 76% (304 Å^2^) for 1a2. Of the total number of contacts (42) that 6-11 makes with the p53R175H, CDR1α, CDR2α, and CDR3α contribute 10%, 0%, and 86%, respectively, compared with 0%, 0%, and 5% by CDR1β, CDR2β, and CDR3β, respectively (Table 2) (Fig. 5, *A*–*C*). The dominance of 6-11 CDR3α in peptide recognition (86% of contacts) exceeds that of 38-10 CDR3α (66%), 1a2 CDR3α (21%), and 12-6 CDR3α (5%) and is only exceeded by 12-6 CDR3β (95%) among all CDR loops in the four TCR–p53R175H–HLA-A2 complexes.

We previously showed that the large majority (∼80%) of contacts between TCRs 38-10, 12-6, and 1a2 and the p53R175H peptide involves C-terminal residues P7 Arg and P8 His, and that these contacts are about evenly distributed between these two residues (19) (Fig. 5, *A*–*C*). These TCRs achieve highly specific recognition of mutant p53 peptide relative to WT by minimizing interactions with the central and N-terminal portions of p53R175H, which are structurally identical in the WT peptide, and instead focusing on the mutation at P8. In sharp contrast to 38-10, 12-6, and 1a2, 6-11 makes no interactions with P8 His (Fig. 5*B*) (Table S4), despite the ability of this TCR to discriminate between mutant and WT p53 (Fig. 1, *A* and *B*). The 6-11–p53R175H–HLA-A2 complex crystallized at pH 8.5. The imidazole group of P8 His should be uncharged at this pH. Instead of P8 His, the principal focus of 6-11 is on the P7 Arg side chain, with which it forms four hydrogen bonds: 6-11 Asp93α Oδ1–Nη2 P7 Arg, 6-11 Asp93α Oδ2–Nη1 P7 Arg, 6-11 Asp93α Oδ2–Nη2 P7 Arg, and 6-11 Pro96α O–Nη2 P7 Arg (Fig. 5*C*). Computational alanine scanning in Rosetta (23) with the 6-11–p53R175H–HLA-A2 complex as input (Table 3) supports the dominance of P7 Arg in 6-11 TCR recognition. In addition, 6-11 Tyr95α makes hydrophobic contacts with P5 Val and P6 Val. However, since P5 Val, P6 Val, and P7 Arg are conserved and highly superimposable in crystal structures of the unbound WT p53–HLA-A2 and mutant p53R175H complexes (19), the mechanism whereby TCR 6-11 distinguishes WT from mutant p53 is not obvious.

**Table 3:** Predicted 6-11 TCR binding affinity changes (ΔΔ*G*s) and component terms for p53 peptide alanine substitutions and WT p53 peptide reversion (H8R)

Peptide substitution	Rosetta ΔΔ*G*^a^	Eatr^b^	Esol^b^	Ehbond^b^
H1A	0	0	0	0
M2A	0	0	0	0
T3A	0.2	0	0	0.2
E4A	0.2	0.6	−0.6	0.2
V5A	0.4	0.5	−0.1	0
V6A	0.6	0.5	0.1	0
R7A	**3.7**	**4.7**	−3.3	**2.3**
H8A	**1.2**	**2**	−0.8	0
C9A	0	0	0	0
H8R	**1.6**	0.2	**1.5**	−0.1

Bold values correspond to largest changes.

a Predicted 6-11 TCR-binding affinity change, calculated by Rosetta (v. 2.3) using the structure of the TCR 6-11–p53R175H–HLA-A2 complex as input.

b Energy term components of the Rosetta ΔΔ*G*. Rosetta terms with values of 0 for all peptide substitutions are not shown. The terms correspond to attractive van der Waals (Eatr), repulsive van der Waals (Erep), desolvation (Esol), and hydrogen bonding (Ehbond) energies.

To resolve this conundrum, we evaluated the effect of replacing P8 His by Arg, which corresponds to reversion to the WT p53 peptide, we carried out *in silico* mutagenesis using Rosetta (23). The peptide substitution was modeled in the X-ray structure of the 6-11–p53R175H–HLA-A2 complex, followed by side-chain minimization and energetics-based scoring to calculate ΔΔ*G*. The predicted ΔΔ*G* value was 1.6 Rosetta energy units (REU; analogous to kcal/mol) (Table 3), consistent with the substantial (60-fold) loss in 6-11 binding affinity for WT p53 peptide that we measured by SPR (Fig. 1*B*). To investigate the mechanistic basis for this affinity loss, the individual Rosetta scoring function terms comprising the predicted ΔΔ*G* were obtained (Table 3). This revealed that the energetic cost of desolvating P8 Arg during complex formation with TCR 6-11 dominated the reduction in binding affinity, contributing 1.5 out of 1.6 REU of the predicted affinity change. Structurally, the limited space around P8 for an Arg residue at that position leads to likely packing interactions of the Arg side chain with the 6-11 TCR CDR loops and its unfavorable desolvation (Fig. S1). The unfavorable effect of desolvating P8 Arg *versus* His by TCR 6-11 is in accordance with the n-octanol to water amino acid transfer energies of Fauchere and Pliska (24), which had an approximately 1.5 kcal/mol hydrophobic energy difference between His and Arg side chains, as well as more recent computed amino acid hydrophobic energies (25) that showed an approximately 2 kcal/mol difference for Arg *versus* His residue desolvation. As with the Rosetta-computed ΔΔ*G* values, these Arg *versus* His amino acid desolvation energy differences are comparable to, albeit slightly less than, the 60-fold binding affinity loss (corresponding to ΔΔ*G* of approximately 2.4 kcal/mol) observed for TCR 6-11 due to the P8 His to Arg substitution. By contrast, a similar previous analysis for TCRs 38-10, 12-6, and 1a2 showed that disruption of hydrogen bonds involving P8 His was mainly responsible for affinity losses of 38-10 and 1a2 for WT p53, while loss of van der Waals interactions accounted for the affinity reduction of 12-6 (19).

We also investigated the energetic contribution of P8 His of p53R175H to binding TCR 6-11 by mutating P8 His to Ala. As noted in Table 3, this substitution was predicted to have a substantial effect on 6-11 TCR binding by Rosetta (23) (ΔΔ*G*: 1.2 REU), which is unexpected, since the 6-11–p53R175H–HLA-A2 structure revealed no interactions between TCR and P8 His using standard cut-off distances of 4.0 Å for van der Waals contacts and 3.5 Å for hydrogen bonds (Table S4). As measured by SPR (Fig. 1*C*), 6-11 bound p53R175A–HLA-A2 with *K*_*D*_ = 98.4 ± 4.2 μM, which is ∼25-fold lower affinity than for p53R175H–HLA-A2 (*K*_*D*_ = 3.5 μM). This destabilization is in accordance with the *in silico* modeling, which likewise predicted less 6-11 binding disruption for P8 His to Ala *versus* His to Arg. In the case of the P8 Ala substitution, the attractive van der Waals term dominated the predicted binding energy loss, indicating that while relatively short-range TCR contacts of the P8 His (<4.0 Å) are not present in the structure, other proximal TCR contacts of the P8 His side chain, for example 6-11 Pro96α, which is <5 Å from P8 His, are favorable interactions that are lost upon Ala substitution. To assess the TCR 6-11 binding impact of additional substitutions at P8, we performed computational mutagenesis to model the effects of all 19 non-His amino acids at that position (Table S5). While certain hydrophobic amino acid residues may allow 6-11 binding, based on this analysis, several charged and polar residues at P8 (*e.g.*, Asp, Gln, and Glu) are predicted to cause major disruptions in 6-11 binding (ΔΔ*G* > 1.0 REU), in addition to Arg and Ala. While computational mutagenesis in Rosetta has been relatively accurate in the context of other TCR–pMHC interfaces (26, 27), due to possible limitations of the Rosetta conformational sampling or scoring function, future experimental binding measurements can confirm these structure-based predictions of hotspots or affinity changes.

## Discussion

During protein–protein complex formation, water molecules are largely excluded from the interface between the interacting partners. The removal of waters exacts a large desolvation penalty that must be offset by attractive hydrophobic and electrostatic contributions in order to form a stable complex. Our computational analysis of the 6-11–p53R175H–HLA-A2 structure revealed that the lower affinity of TCR 6-11 for WT p53–HLA-A2 (*K*_*D*_ = 236 μM) than for mutant p53R175H–HLA-A2 (3.8 μM) is primarily due to the higher energetic cost of desolvating P8 Arg in the WT p53 peptide than P8 His in the mutant. Importantly, this unusual strategy for distinguishing WT from mutant p53 does not rely on direct contacts between TCR 6-11 and P8 His, in marked contrast to the more typical strategies employed by TCRs 38-10, 12-6, and 1a2, which depend on direct contacts.

Although we do not observe direct contacts between TCR 6-11 and P8 His, we cannot rule out indirect interactions mediated by bound water molecules. The limited resolution of the 6-11–p53R175H–HLA-A2 structure (3.33 Å) does not permit the identification of ordered waters with confidence, and none were included in the final model. However, in several high resolution TCR–pMHC structures (≤2.5 Å), interfacial waters have been found to form bridging hydrogen bonds that enhance polar interactions and neutralize unpaired hydrogen-bonding groups (28).

In addition to TCRs, antibodies are also under active investigation for immunotherapeutic targeting of cancer neoantigens. A monoclonal antibody (H2) specific for p53R175H–HLA-A2, the exact same pMHC targeted by TCR 6-11, was recently reported (29). In the crystal structure of Fab H2 bound to p53R175H–HLA-A2, the V_L_CDR3 and V_H_CDR1–3 loops form a tight cage around P7 Arg and P8 His in which the imidazole side chain of P8 His is part of a hydrogen bonding network with V_L_CDR3 Tyr94 and V_H_CDR2 Asp54. Thus, antibody H2, like TCRs 38-10, 12-6, and 1a2, distinguish WT from mutant p53 *via* direct contacts with P8 His, which is fundamentally different from the indirect strategy utilized by TCR 6-11.

## Experimental procedures

### Protein preparation

The isolation and characterization of p53R175H-specific TCR 6-11 from patients with colorectal cancer was described previously (14). Soluble TCR 6-11 for affinity measurement and structure determination was produced by *in vitro* folding from inclusion bodies expressed in *E. coli*, as described previously for other p53R175H-specific TCRs (19). Codon-optimized genes encoding the TCR α (residues 1–205) and β (residues 1–245) chains were synthesized and cloned into the expression vector pET22b (GenScript). An interchain disulfide (CαCys159–CβCys172) was engineered to increase the folding yield of TCR αβ heterodimer. Disulfide-linked TCR 6-11 was purified using sequential Superdex 200 and MonoQ FPLC columns (GE Healthcare).

Soluble HLA-A2 loaded with WT p53 peptide (HMTEVVRRC) or mutant p53R175H peptide (HMTEVVRHC) was prepared by *in vitro* folding of *E. coli* inclusion bodies as described (19). Correctly folded p53–HLA-A2 and p53R175H–HLA-A2 were purified using consecutive Superdex 200 and MonoQ columns. To produce biotinylated HLA-A2, a C-terminal tag (GGGLNDIFEAQKIEWHE) was attached to the HLA-A2∗0201 heavy chain. Biotinylation was carried out with BirA biotin ligase (Avidity).

### Crystallization and data collection

For crystallization of the TCR 6-11–p53R175H–HLA-A2 complex, TCR 6-11 was mixed with p53R175H–HLA-A2 in a 1:1 M ratio at a concentration of 14 mg/ml. Crystals were obtained at room temperature by vapor diffusion in hanging drops. The 6-11–p53R175H–HLA-A2 complex crystallized in 20% (w/v) PEG 3350, 0.1 M Bis-Tris propane (pH 8.5), and 0.2 M potassium thiocyanate. For data collection, the crystals were cryoprotected with 20% (w/v) glycerol and flash-cooled. X-ray diffraction data were collected at beamline 23-ID-D of the Advanced Photon Source, Argonne National Laboratory. The diffraction data were indexed, integrated, and scaled using the program HKL2000 (30). Data collection statistics are shown in Table S1.

### Structure determination and refinement

Data reduction was performed using the CCP4 software suite (31). The TCR 6-11–p53R175H–HLA-A2 structure was solved by molecular replacement with the program Phaser (32) and refined by Phenix with NCS constraints (33). The model was further refined by manual model building with Coot (34) based on 2*F*_o_ – *F*_c_ and *F*_o_ – *F*_c_ maps. The α chain of a CD1b-specific TCR (PDB accession code 6OVN) (35), the β chain of dengue virus-specific TCR D30 (5WKF) (36), and p53R175H–HLA-A2 (6VR5) (19) with the CDRs and peptide removed were used as search models to determine the orientation and position of the 6-11–p53R175H–HLA-A2 complex. Refinement statistics are summarized in Tables S1. Contact residues were identified with the CONTACT program (31) and were defined as residues containing an atom 4.0 Å or less from a residue of the binding partner. Figures were prepared using PyMOL (https://pymol.org/).

### Surface plasmon resonance analysis

The interaction of TCR 6-11 with p53–HLA-A2 and p53R175H–HLA-A2 was assessed by SPR using a BIAcore T100 biosensor. Biotinylated p53–HLA-A2 or p53R175H–HLA-A2 was immobilized on a streptavidin-coated BIAcore SA chip (GE Healthcare) at 3000 resonance units (RU). An additional flow cell was injected with free biotin alone to serve as a blank control. For the analysis of TCR binding, solutions containing different concentrations of 6-11 were flowed sequentially over chips immobilized with p53–HLA-A2, p53R175H–HLA-A2, or the blank. The time point before the ending injections was used as the equilibrium level. Dissociation constants were calculated by fitting equilibrium and kinetic data to a 1:1 binding model using BIA evaluation 3.1 software.

### Computational structural analysis

Calculation of TCR–pMHC crossing and incident angles was performed as previously described (19). Computational mutagenesis to predict 6-11 TCR binding affinity changes (ΔΔ*G*) was performed using a ΔΔ*G* prediction protocol in Rosetta version 2.3 (23), which was previously used to predict affinity changes of a therapeutic TCR–peptide–MHC interface (27), and was shown to be effective for ΔΔ*G* and hotspot prediction for antibody–antigen interfaces (37). Local side chain minimization was performed before and after in silico mutagenesis in Rosetta, as specified with a command line parameter (“-minint_intchi”). A sample command line is:

rosetta.gcc aa 611.pdb _ -interface -intout 611.ddg.ros.out -skip_missing_residues -mutlist 611.muts.txt -min_interface -int_chi -extrachi_cutoff 1 -ex1 -ex2 -ex3 -s 611.pdb

### Calculation of TCR centers

Calculations of TCR center positions were performed as described previously (19). The 6-11–p53R175H–HLA-A2 complex was translated and rotated into a reference frame used in our previous study (19), with the MHC helix plane aligned to the *x*–*y* plane. This reoriented complex was then used to calculate the TCR variable domain center and its projection onto the *x*–*y* plane, giving its *x* position and *y* position over the centered and oriented MHC in Ångstrom units. These positions were compared with values calculated for other structurally characterized TCR–peptide–MHC class I complexes in that same reference frame, obtained from the TCR3d database (38); all positions except the 6-11 TCR position were reported in our recent study (19).

## Data availability

Atomic coordinates and structure factors for the TCR 6-11–p53R175H–HLA-A2 complex have been deposited in the Protein Data Bank under accession code 7RM4.

## Supporting information

This article contains supporting information (31)

## Conflict of interest

The authors declare no competing interests.
