# Supplementary material for: T cell receptors employ diverse strategies to target a p53 cancer neoantigen
Source: J Biol Chem. 2022 Feb 3;298(3):101684. doi: 10.1016/j.jbc.2022.101684 (PMC8897694; doi:10.1016/j.jbc.2022.101684)
Supplement: Supporting Tables S1–S5 and Figure S1 [file mmc1.pdf]

# Supporting Information

## T cell receptors (TCRs) employ diverse strategies to target a p53 cancer neoantigen

Daichao Wu, Ragul Gowathaman, Brian G. Pierce, and Roy A. Mariuzza

**Supporting Tables 1–5**

**Supporting Figure 1**

**Table S1. Data collection and structure refinement statistics**

| | **TCR 6-11–p53R175H–HLA-A2** |
|---|---|
| PDB accession code | 7RM4 |
| **Data collection** | |
| Resolution range (Å)[a] | 49.3–3.33 (3.45–3.33) |
| Space group | *P*1211 |
| Unit cell parameters | 127.2 Å, 55.1 Å, 304.9 Å<br>90°, 98.7, 90° |
| Total reflections[a] | 294,644 (33,281) |
| Unique reflections[a] | 60,837 (4,526) |
| Multiplicity[a] | 4.8 (5.3) |
| Completeness (%)[a] | 92.6 (72.0) |
| Mean $I/\sigma(I)$[a] | 10.3 (4.0) |
| Wilson *B* factor (Å$^2$) | 59.5 |
| $R_{merge}$[a,b] | 0.169 (0.730) |
| CC1/2 | 0.992 (0.737) |
| | |
| **Refinement** | |
| Resolution range (Å) | 49.3–3.33 |
| Reflections used in refinement[a] | 58,081 (4,525) |
| $R_{work}$[c] | 0.237 (0.322) |
| $R_{free}$[c] | 0.293 (0.387) |
| No. of protein atoms | 26,474 |
| Protein residues | 3308 |
| r.m.s.d. from ideality | |
| Bond lengths (Å) | 0.006 |
| Bond angles (°) | 0.92 |
| Ramachandran plot statistics | |
| Favored (%) | 92.2 |
| Allowed (%) | 7.0 |
| Disallowed (%) | 0.7 |
| Rotamer outliers (%) | 1.0 |
| Clashscore | 9.8 |
| Average *B* factor (Å$^2$) | 50.6 |
| Protein | 50.6 |

[a]Values in parentheses correspond to the highest resolution shell.

[b]$R_{merge} = \sum|I_j - \langle I \rangle|/\sum I_j$, where $I_j$ is the intensity of an individual reflection and $\langle I \rangle$ is the average intensity of that reflection.

[c]$R_{work}$ ($R_{free}$) = $\sum||F_o| - |F_c||/\sum|F_o|$; 5.0% of data were used for $R_{free}$.

**Table S2. TCR center positions over peptide-MHC plane for MHC class I complexes**

| Complex[1] | x pos[2] | y pos[2] |
|---|---|---|
| 5SWS | 22.3 | -14.8 |
| 5SWZ | 22.3 | -13.9 |
| 38-10 | 14.8 | -6.1 |
| 3TJH | 13.8 | -3.3 |
| 5TEZ | 13 | -0.8 |
| 3TF7 | 12.9 | 3.0 |
| 6-11 | 12.8 | -3.7 |
| 3PQY | 12.5 | 1.6 |
| 4G9F | 12.5 | -4.8 |
| 3TFK | 12.4 | 2.5 |
| 4G8G | 12.2 | -4.6 |
| 3KPR | 11.8 | -1.4 |
| 3KPS | 11.8 | -1.3 |
| 4N0C | 11.3 | 1.6 |
| 4N5E | 11.3 | 1.8 |
| 5IVX | 10.9 | 1.6 |
| 1MI5 | 10.6 | -2.8 |
| 2OL3 | 10.6 | 0.1 |
| 5W1W | 10.4 | -0.5 |
| 4MXQ | 10.3 | 2.9 |
| 1NAM | 10 | -1.8 |
| 5W1V | 10 | -0.6 |
| 12-6 | 10.0 | -2.3 |
| 1FO0 | 9.6 | -0.4 |
| 5D2L | 8.8 | -1.7 |
| 3TPU | 8.1 | 0.1 |
| 1a2 | 8.1 | -0.3 |
| 4MS8 | 8 | 1.8 |
| 3GSN | 7.6 | -3.2 |
| 2OI9 | 7.4 | 2.2 |
| 5M01 | 7.4 | -2.2 |
| 6G9Q | 7.4 | -2.2 |
| 5M00 | 7.2 | -1.8 |
| 5D2N | 7.1 | -2.5 |
| 5HHO | 7.1 | 4.9 |
| 3E3Q | 7 | 2 |
| 5EUO | 7 | 4.6 |
| 3E2H | 6.9 | 1.8 |
| 2E7L | 6.8 | 1.9 |
| 4MVB | 6.8 | 1.2 |
| 5M02 | 6.8 | -2.1 |

| | | |
|---|---|---|
| 5TIL | 6.6 | -2.1 |
| 2VLR | 6.5 | 4.5 |
| 5HHM | 6.5 | 4.7 |
| 5E6I | 6.4 | 6.5 |
| 5TJE | 6.4 | -2.2 |
| 2ESV | 6.3 | 2.4 |
| 3SJV | 6.2 | -2.5 |
| 1G6R | 6 | 1.2 |
| 1MWA | 5.9 | 1.5 |
| 2BNQ | 5.9 | -1.9 |
| 1OGA | 5.8 | 4 |
| 2PYE | 5.8 | -3.6 |
| 4EUP | 5.8 | 1.3 |
| 5EU6 | 5.8 | 1.1 |
| 6MTM | 5.8 | 1 |
| 2BNR | 5.7 | -2.1 |
| 2P5E | 5.7 | -3.5 |
| 2P5W | 5.6 | -3.5 |
| 5YXU | 5.6 | -2.7 |
| 2YPL | 5.5 | 1.2 |
| 4MJI | 5.4 | 1.9 |
| 5WLG | 5.3 | 1.5 |
| 5JZI | 5.2 | -1.7 |
| 2F53 | 5 | -3 |
| 3QDM | 4.9 | -2.3 |
| 5E9D | 4.8 | 5.3 |
| 5ISZ | 4.6 | 3.9 |
| 5JHD | 4.5 | 4.9 |
| 6BJ2 | 4.4 | 5.6 |
| 5MEN | 4.1 | 5.1 |
| 1LP9 | 4 | 2.7 |
| 4NHU | 4 | -5.7 |
| 4MNQ | 3.9 | 5.2 |
| 3DXA | 3.6 | -1.5 |
| 3RGV | 3.4 | -1.2 |
| 4PRH | 3.4 | 3 |
| 3MV7 | 3.1 | 2.4 |
| 3MV8 | 3.1 | 2.6 |
| 3MV9 | 3.1 | 2.7 |
| 4PRP | 3 | 2.6 |
| 3VXM | 2.7 | -3.4 |
| 5NHT | 2.7 | 1.1 |
| 5NQK | 2.4 | 0.9 |

| | | |
|---|---|---|
| 5NMG | 2.2 | 3.2 |
| 3VXS | 2.1 | -0.6 |
| 5C0B | 2.1 | -1.9 |
| 3QEQ | 2 | -0.8 |
| 6AVG | 2 | 1.2 |
| 3O4L | 1.7 | -1.2 |
| 5C0C | 1.7 | -1.7 |
| 5NME | 1.7 | 3.1 |
| 6D78 | 1.7 | -0.2 |
| 4L3E | 1.6 | -0.4 |
| 3VXR | 1.5 | -0.6 |
| 5C08 | 1.4 | -2.8 |
| 5C0A | 1.4 | -0.8 |
| 3QDG | 1.3 | -1 |
| 5C09 | 1.3 | -1.5 |
| 5WKF | 1.3 | -1.3 |
| 6DKP | 1.3 | -0.2 |
| 2AK4 | 1.2 | 6.6 |
| 3QDJ | 1.2 | -0.9 |
| 5C07 | 1.2 | -1.7 |
| 5NMF | 1.2 | 3.2 |
| 5WKH | 1.2 | -1.6 |
| 3VXU | 1.1 | 3.3 |
| 5HYJ | 1 | -2.3 |
| 6AM5 | 0.9 | -0.8 |
| 4JRX | 0.8 | 8.5 |
| 1KJ2 | 0.7 | 2.5 |
| 2GJ6 | 0.6 | 1.8 |
| 3UTS | 0.4 | -0.2 |
| 6EQA | 0.2 | -1.5 |
| 4JFD | 0.1 | -2.5 |
| 5BRZ | 0.1 | 6.4 |
| 5BS0 | 0.1 | 6.8 |
| 3QFJ | 0 | 1.7 |
| 6EQB | 0 | -2.2 |
| 3PWP | -0.1 | 1.6 |
| 4JFE | -0.1 | -2.4 |
| 4JFF | -0.1 | -2.4 |
| 4QOK | -0.1 | -1.8 |
| 6AMU | -0.1 | 0.2 |
| 1QRN | -0.2 | 2 |
| 3H9S | -0.2 | 2 |
| 3HG1 | -0.2 | -1.9 |

| | | |
|---|---|---|
| 4FTV | -0.3 | 1.6 |
| 1AO7 | -0.4 | 2 |
| 1QSE | -0.5 | 1.4 |
| 2NX5 | -0.7 | 2.4 |
| 1QSF | -0.8 | 1.5 |
| 4QRP | -0.9 | -1.3 |
| 1BD2 | -1.3 | -0.3 |
| 6AVF | -1.7 | -0.1 |
| 3FFC | -2.3 | 2.9 |
| 4JRY | -14.6 | 0.8 |

[1]PDB code for complex structure, with four TCR–p53R175H–HLA-A2 complex structures given by TCR name (6-11, 12-6, 38-10, 1a2) and corresponding rows highlighted.

[2]TCR–pMHC complexes were oriented into a common reference frame centered at average Cα atom position of MHC helices, and rotated such that the *x*–*y* plane is parallel with the helix plane, and the *x*-axis is parallel to peptide groove, with greater *x* value corresponding to peptide C-terminus. All values are in Ångstrom units. TCR variable domain centers were calculated by taking centers of individual variable domains by average positions of Sγ atoms of conserved Cys residues (or Cα atoms at corresponding positions where Cys residues are not present in the TCR), and then calculating the mean position of TCR Vα and Vβ centers. *X* position (*x* pos) and *y* position (*y* pos) values represent projections into the *x*–*y* plane, and thus the MHC helix plane, of these centers.

## Table S3. Interactions between TCRs and HLA-A2

| HLA-A2 | TCR 6-11 | | TCR 38-10 | | TCR12-6 | | TCR 1a2 | |
|---|---|---|---|---|---|---|---|---|
| | Hydrogen bonds | Van der Waals contacts | Hydrogen bonds | Van der Waals contacts | Hydrogen bonds | Van der Waals contacts | Hydrogen bonds | Van der Waals contacts |
| α1 | | | | | | | | |
| R65H | | | | S28α(2) | G94α(O) R65H(Nη2)<br>Q96α(Nε2) R65H(Nε) | G94α(7)<br>G93α(1)<br>Y95α(1)<br>Q96α(9) | L94α(O) R65H(Nη2) | A29α(3)<br>L94α(3)<br>E96α(2) |
| K68H | | | | | | Y95α(2) | E96α(O) K68H(O) | E96α(4) |
| A69H | | | | S98α(1) | | Y95α(5)<br>W98β(4) | | L94α(3)<br>K95α(1)<br>S98α(1) |
| Q72H | | | | | | Y95α(3)<br>W98β(9) | | E96α(1)<br>D97α(5)<br>S98α(2) |
| T73H | | P96α(2) | | | | W98β(2) | | S98α(1) |
| R75H | V54β(O) R75H(Nη1)<br>V54β(O) R75H(Nη2)<br>V55β(O) R75H(Nη1) | V54β(4)<br>V55β(2) | | D56β(1) | N30β(Oδ1) R75H(Nη2)<br>S51β(Oγ) R75H(Nη1) | N30β(2)<br>S51β(2) | | |
| V76H | | | | Y50β(1) | | N30β(1) | | |
| T80H | | | | R30β(2) | | | | |
| α2 | | | | | | | | |
| K146H | | D97β(4) | R30β(Nη2) K146H(Nζ) | R30β(2)<br>L96β(2) | | | | |
| A149H | D100β(N) A149H(O)<br>D100β(Oδ1) A149H(O) | Y32α(1)<br>G99β(2)<br>D100β(2) | | L96β(1) | | | | D100β(1) |
| A150H | | Y32α(4)<br>P98β(1)<br>G99β(1) | Y97α(Oη) A150H(O) | Y97α(5) | | Y51α(5) | | |
| H151H | R51α(Nη1) H151H(Nδ1)<br>R51α(Nη1) H151H(Nε2) | Y32α(1)<br>R51α(11)<br>D100β(4) | E52α(Oε2) H151H(Nδ1)<br>K55α(Nζ) H151H(Nε2) | E52α(5)<br>Y54α(1)<br>K55α(5) | | Y51α(18) | | Y51α(2) |
| V152H | | | | Y97α(3) | | | | |
| E154H | N53α(Nδ2) E154(Oε1)<br>N53α(Nδ2) E154(Oε2) | N53α(5) | | Y54α(3) | S52α(N) E154(Oε1)<br>S53α(Oγ) E154(Oε1)<br>S53α(Oγ) E154(Oε2)<br>S53α(N) E154(Oε1) | Y51α(4)<br>S52α(3)<br>S53α(6) | | Y51α(4) |
| Q155H | Y95α(Oη) Q155H(Oε2) | A31α(2)<br>Y95α(4) | N31α(Nδ2) Q155H(Oε1)<br>N31α(Nδ2) Q155H(Nε2)<br>Y97α(Oη) Q155H(Nε2) | N31α(3)<br>Y54α(2)<br>Y97α(3) | S32α(Oγ) Q155H(Oε1)<br>Q31α(Nε2) Q155H(Oε1) | Q31α(3)<br>Y51α(1)<br>V100β(2) | Q31α(Oε1) Q155H(Nε2)<br>Y32α(Oη) Q155H(Oε1)<br>Y32α(Oη) Q155H(Nε2) | Y32α(7)<br>Y51α(6) |

Contact residues were identified with CONTACT (31). Hydrogen bonds were calculated using a cut-off distance of 3.5 Å. The cut-off distance for van der Waals contacts was 4.0 Å.

## Table S4. Interactions between TCRs and p53R175H peptide

| p53R175H | TCR 6-11 | | TCR 38-10 | | TCR 12-6 | | TCR 1a2 | |
|---|---|---|---|---|---|---|---|---|
| | Hydrogen bonds | Van der Waals contacts | Hydrogen bonds | Van der Waals contacts | Hydrogen bonds | Van der Waals contacts | Hydrogen bonds | Van der Waals contacts |
| E4p | | S29α(3)<br>Y95α(5) | S98α(Oγ) E4p(O)<br>N30α(N) E4p(Oε2) | E29α(3)<br>N30α(8)<br>S98α(3) | G94α(N) E4p(Oε1) | V100β(1)<br>G93α(2) | Y100α(Oη) E4p(O) | A29α(6)<br>Q31α(1)<br>L94α(2)<br>Y100α(3) |
| V5p | | Y95α(10) | | Y97α(1) | | | | Y100α(1) |
| V6p | | Y95α(4)<br>P96α(2) | | S98α(1) | V100β(N) V6p(O) | W98β(1)<br>Q99β(3)<br>V100β(1) | Q97β(Nε2) V6p(O) | L94α(1)<br>Y100α(2) |
| R7p | D93α(Oδ1) R7p(Nη2)<br>D93α(Oδ2) R7p(Nη1)<br>D93α(Oδ2) R7p(Nη2)<br>P96α(O) R7p(Nη2) | P98β(2)<br>Y32α(1)<br>D93α(5)<br>Y95α(3)<br>P96α(3) | Y97α(O) R7p(Nη2)<br>L96β(O) R7p(Nη1)<br>V97β(O) R7p(Nη1) | Y97α(10)<br>Y103α(12)<br>V97β(1)<br>T98β(1) | Q99β(Oε1) R7p(Nε)<br>V100β(O) R7p(Nη2)<br>G101β(O) R7p(Nη2)<br>E103β(Oε1)<br>R7p(Nη1) | Q99β(12)<br>V100β(3)<br>G101β(2)<br>E103β(5) | D100β(Oδ2) R7p(Nη1)<br>Y32α(Oη) R7p(Nη1)<br>D100β(Oδ2) R7p(Nη2) | Q96β(2)<br>Q97β(4)<br>A99β(3)<br>D100β(2)<br>Y32α(3)<br>Y100α(1) |
| H8p | | | Y103α(Oη) H8p(N)<br>Y31β(Oη)<br>H8p(Nδ1) | Y31β(5)<br>R30β(3)<br>Y50β(1)<br>Y103α(24) | E95β(Oε2) H8p(Nδ1)<br>W98β(Nε1) H8p(Nε2)<br>Q99β(Oε1) H8p(N) | E95β(5)<br>G96β(2)<br>W98β(3)<br>Q99β(10) | Q97β(Oε1) H8p(N)<br>Q96β(Nε2) H8p(O)<br>S98α(Oγ) H8p(Nε2) | M50β(1)<br>Q96β(4)<br>Q97β(15)<br>S98α(2) |
| C9p | | | R30β(Nη2) C9p(O) | R30β(1) | | | | |

Contact residues were identified with CONTACT (31). Hydrogen bonds were calculated using a cut-off distance of 3.5 Å. The cut-off distance for van der Waals contacts was 4.0 Å.

**Table S5. Predicted TCR 6-11 affinity changes (ΔΔ*G*s) for substitutions at P8 of peptide p53R175H**

| Peptide substitution | Rosetta ΔΔ*G*[1] |
|---|---|
| H8A | **1.2** |
| H8C | **1.2** |
| H8D | **1.3** |
| H8E | **1.1** |
| H8F | -0.7 |
| H8G | **1.3** |
| H8I | 0.7 |
| H8K | 0.6 |
| H8L | 0.6 |
| H8M | 0.1 |
| H8N | **1.3** |
| H8P | 0.7 |
| H8Q | **1.2** |
| H8R | **1.6** |
| H8S | **1.3** |
| H8T | 1.0 |
| H8V | 1.0 |
| H8W | -1.0 |
| H8Y | -0.8 |

[1]Predicted TCR 6-11 binding affinity change, calculated by Rosetta (v. 2.3) using the 6-11–p53R175H–HLA-A2 complex structure as input. Values are in Rosetta Energy Units (REU) and analogous to energies in kcal/mol. Values in bold correspond to substantial predicted affinity disruptions (>1.0 REU).

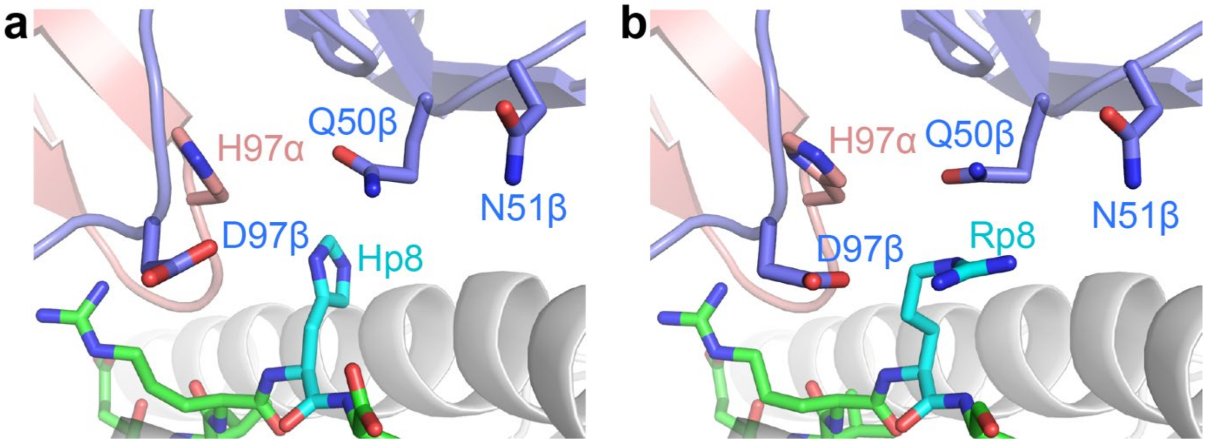


**Figure S1. Modeled peptide residue P8 (p53 residue 175) packing for arginine reversion substitution.** Structure of TCR 6-11 in complex with (*a*) p53R175H–HLA-A2, from X-ray structure, and (*b*) p53R175–HLA-A2, with peptide reversion arginine substitution modeled using Rosetta. Peptide shown as green sticks, with peptide residue P8 cyan. HLA-A2 is shown as gray cartoon, and TCR α and β chains, pink cartoon and blue cartoon, respectively. TCR residues proximal to P8 residue are labeled and shown as sticks.
